# Should We Use Behavioural Predictions in Organ Allocation?

**DOI:** 10.1111/bioe.13440

**Published:** 2025-06-29

**Authors:** Max Drezga-Kleiminger, Dominic Wilkinson, Thomas Douglas, Joanna Demaree-Cotton, Julian Koplin, Julian Savulescu

**Affiliations:** 1 Faculty of Medicine, Nursing and Health Sciences, Monash University, Melbourne, Victoria, Australia; 2 Oxford Uehiro Centre for Practical Ethics, Faculty of Philosophy, University of Oxford, Oxford, UK; 3 Centre for Biomedical Ethics, Yong Loo Lin School of Medicine, National University of Singapore, Singapore, Singapore; 4 John Radcliffe Hospital, Oxford, UK; 5 Monash Bioethics Centre, Monash University, Melbourne, Victoria, Australia; 6 Murdoch Children's Research Institute, Melbourne, Victoria, Australia

**Keywords:** artificial intelligence, behaviour, ethics, liver allocation, medicine, predictions, resource allocation

## Abstract

Medical predictions, for example, concerning a patient's likelihood of survival, can be used to efficiently allocate scarce resources. Predictions of patient behaviour can also be used—for example, patients on the liver transplant waiting list could receive lower priority based on a high likelihood of non-adherence to their immunosuppressant medication regimen or of drinking excessively. But is this ethically acceptable? In this paper, we will explore arguments for and against behavioural predictions, before providing novel empirical evidence on this question. Firstly, we note that including behavioural predictions would lead to improved transplant outcomes. Fairness could also require prioritising those predicted to engage in healthier behaviours: consistent with using behavioural predictions in other contexts such as psychiatry and substance misuse. Conversely, behavioural predictions may be judged too inaccurate or discriminatory, or it may be thought unfair to deprioritise based on future behaviour. In part two, we performed an online survey of 172 UK adults. When presented with possible factors relevant to liver allocation, most thought predictions of higher medication adherence (78.6%) and lower future alcohol use (76.5%) should be used but not predictions of lower future criminality (24.7%) and higher societal contribution (21.2%). Randomising participants into two groups, 69.8% of participants found deprioritising a patient based on their predicted medication adherence acceptable (91.9% found a nonbehavioural prediction acceptable). We did not identify an ethically relevant difference between behavioural predictions and other medical predictions already used in organ allocation. Our sample of participants also appeared to support behavioural predictions in this context.

## 1 Background

Treating patients differently based on predictions about them is common. Emergency departments prioritise those predicted to suffer the most harm without treatment: this is the basis of triage. Cancer treatment is recommended based on the likelihood and rate of predicted spread. When resources are limited, treatment may also be allocated based on predictions. For example, patients awaiting liver transplantation are often prioritised based on predicted urgency (how soon death is predicted to occur without a transplant), utility, or benefit of a transplant [1]. In this paper we will refer to ‘utility’ as a patient's predicted survival if they were to receive a transplant, and ‘benefit’ as the predicted net benefit (i.e., difference in predicted survival with and without transplant), consistent with the terminology used in the UK NHS transplant guidelines [2]. Predictions have historically relied upon clinician experience, data-based models, or algorithms that consider various medical variables [1]. Recently, machine learning AI has been proposed to improve the accuracy of these predictions [3].

Aside from purely medical predictions, decisions about treatment are also sometimes influenced by predictions of patients' behaviour. Examples include the use of violence or self-harm risk assessments in psychiatry and predictions of patients' risk of misusing opioids [4–6]. Behavioural predictions may also be relevant to allocation of organs for transplantation [7]. For example, assessing risk of substance abuse or medical non-adherence could improve predictions of medical gains from transplantation. More controversial examples could include predicting nonmedical outcomes, such as the likelihood of the recipient committing a crime or making significant contributions to society.

Some behavioural predictions may already be considered by transplant teams as reasons against listing a patient for transplantation. UK liver transplantation guidelines state:

A multidisciplinary approach is required to select patients who are likely to comply with follow-up and not return to a damaging pattern of alcohol consumption after transplantation. [8]

Australian guidelines likewise suggest there may be a threshold at which a transplant is considered worthwhile—that is, individuals who are deemed at a ‘high risk’ of behaviours which could endanger a donor organ are excluded from the waiting list [9]. More controversially, patients could also be ranked or downgraded according to predicted future actions, even after meeting eligibility requirements. But is using predicted behaviour ethically more problematic than using predicted medical outcomes (e.g., predicted survival) in allocating livers for transplantation?

One way to distinguish medical and behavioural predictions would be to appeal to individual control. Individuals typically have control over their behaviour, whereas more commonly used ‘medical’ predictions might concern events, such as a patient’s intrinsic immunological rejection of an organ, over which the patient often has little or no control.^1^ We will use the term ‘natural events’ (and ‘natural predictions’) to refer to the latter category, and ‘behaviour’ (and ‘behavioural predications’) to refer to the former. However, control exists on a spectrum. For instance, alcohol addiction may influence drinking behaviour in ways partially outside an individual’s autonomous control.

For simplicity, and to best draw out the relevant ethical issues, we focus on the two ends of the spectrum: natural events that are (almost) entirely beyond a person's control, and behaviours that are (almost) entirely within their control.

In part 1 of this paper, we introduce potential arguments that could justify behavioural predictions. We begin with outcome-based and fairness-based justifications, explore their parallels in other areas of medicine, and examine whether there are morally relevant differences between natural and behavioural predictions. (If there are moral differences, it would then be important to consider how to respond to borderline or mixed cases.)

Our analysis focuses on liver transplantation, though the arguments may apply to other organs and scarce resources. Consistent with current practice, we assume that it is ethically acceptable to include natural predictions in allocation. (If someone were to hold that organs should be allocated on the basis of first-come-first served or a lottery, they would likely reject the use of both natural and behavioural predictions.) It is likely that behavioural predictions, if adopted, would be only one factor in allocation that is weighed alongside other considerations. However, for the sake of simplicity, and given the dearth of existing research on this topic, the discussion below focuses on whether they should be used at all—not how much weight they should be granted relative to other criteria.

Public attitudes are also critical. Even if there are no intrinsic ethical objections to using behavioural predictions, it would be important to understand whether the public views matters differently. First, for a health policy to be legitimate in a democratic society, public support is necessary [10]. Second, public opinion on organ allocation policy may affect willingness to donate, which is practically relevant to the supply of organs [11].

Previous research on public attitudes in criminal justice indicate ambivalence towards the use of algorithms predicting behaviour [12], and these continue to be contentious [13, 14]. We are not aware of previous work assessing public attitudes towards predicting behaviour in medicine. In part 2, we report a small pilot study investigating the views of a sample of lay-people on the use of natural and behavioural predictions. We conclude by integrating the ethical and empirical findings, ultimately defending the use of behavioural predictions in this context.

## 2 Part 1: Ethical Analysis

Consider the following patients with liver failure (Box 1). Imagine that they are similar in all other respects. A single liver is available that would be suitable for either patient. Should they be treated differently for the purposes of allocating a liver?

### 2.1 Maximising Benefit

The main advantage of using behavioural predictions in liver allocation would be to maximise the utility or benefit produced by a scarce resource, given the chronic shortage of organs worldwide [1].

Outcomes have always been considered an important principle in resource allocation; however, these have been difficult to implement in policy, at least in part due to predictive uncertainty—it is difficult to make confident predictions about survival following treatment in complex contexts such as transplantation [1]. Subsequently, most countries, such as Australia, have traditionally relied upon MELD score (a basic algorithm used to predict waiting list survival, i.e., urgency) or its modifications as the basis for selecting patients for liver transplantation [9]. Where outcome predictions have been used, they have been simple, e.g. in Australia, recipients are matched by blood type, a basic measure aimed at improving transplant success. However, since 2018, the United Kingdom has started allocating livers using a newly developed outcome-based algorithm—the Transplant Benefit Score (TBS). The TBS uses 21 recipient criteria and seven donor criteria to predict the net survival benefit of a transplant for each patient [8].

The use of more sophisticated algorithms may allow us to make more accurate and consistent predictions, and therefore could make implementing outcome into organ allocation policy more feasible [1]. For this reason, the TBS has garnered much interest from the international transplantation community, particularly from those who have argued that prioritarianism-based (e.g., based on MELD score) allocation leads to an unacceptable waste of precious organs [15]. Since implementation of the TBS in the United Kingdom, preliminary evidence indicates that waiting list mortality has fallen and post-transplant survival remained approximately the same—leading to an overall net benefit [16].^2^ The algorithm is being refined further, which may lead to ongoing improvements.

In beginning to use outcome predictions in liver allocation, we must also consider the relevance of behaviours which influence outcomes, such as immunosuppressant adherence and substance use. For instance, alcohol relapse is the strongest available predictor of post-transplant mortality [17]. Would we also accept predictions of these behaviours as variables integrated into the TBS or another outcome-sensitive allocation policy? To a certain extent, this may already implicitly be the case. Depending on how it is developed, an algorithm predicting survival may ‘accidentally’ make associations between its input variables and behaviour. For example, some variables in the TBS already act as implicit proxies for behaviour. For example, ‘hepatitis C status’ could reflect both biological risk (e.g., viral relapse [18]) and behavioural associations like intravenous drug use. Including this variable may, therefore, actually involve selecting against those who are likely to inject drugs in the future. Furthermore, non-causative associations could also be made between other behaviours and this variable. For example, there is also an association between hepatitis C status and alcohol use [19]; therefore, this variable may also be a proxy for future alcohol consumption. It may therefore be difficult to separate natural and behavioural components of outcome predictions. Whether this is important—and therefore efforts should be made to disentangle the two—depends on whether there is an ethically relevant difference between these.

Led by the United Kingdom, the trend is towards an increased use of predicted benefit in allocation decisions, although exactly how this should be weighed against other factors, such as urgency, is a topic of debate [20, 21]. If we accept that outcome is (at least in part) relevant and that behaviour affects outcomes, predicting behaviour would also be ethically desirable in liver allocation, unless some ethically relevant distinction can be found between behavioural and natural predictions.

### 2.2 Future Responsibility, Fairness and Autonomy

A potential reason to consider *past* behaviour, such as alcohol use, when allocating livers is the view that these patients are in some way morally responsible for their liver disease. Individuals are sometimes argued to be morally responsible if they fulfil epistemic and control conditions, that is, they know that alcohol may lead to liver disease, and their chronic drinking is a voluntary behaviour which they can control [22]. If they are responsible in this way, their drinking may weaken their claim to receive a transplant, compared to others who are not responsible for their liver failure. Several responsibility-sensitive approaches may justify using past behaviour to deprioritise patients [23, 24]. For example, luck egalitarian approaches prioritise patients whose illness results from chance over those whose choices contributed to their condition [25, 26].

However, using past patient behaviour and responsibility for illness in organ allocation is controversial. Identifying responsibility is challenging. For example, there are many factors that are outside of an individuals' control that contribute to alcohol consumption, and addiction is often considered to diminish responsibility by undermining the voluntariness of the behaviour [27]. Even if we agree to hold an individual culpable for drinking, it can also be difficult to establish a causal link between the behaviour and the illness. There are other genetic and epigenetic factors which may contribute to the development of liver disease to varying degrees, which are clearly beyond the control of the individual.

Additionally, health is often seen as a special good that should be distributed based on medical need rather than desert—unlike areas such as criminal justice [28, 29]. Proponents of this idea argue that health can be seen as a fundamental moral good, which affords individuals with opportunities which should be protected, or that health-related harms outweigh other harms. It may also be argued that outcomes of these policies are too harsh [30]—the ‘punishment’ of being denied a liver-saving liver transplant may be disproportionate to the ‘crime’ of consuming alcohol. Furthermore, because the behaviours that these policies target can be socially determined, responsibility-sensitive policy may adversely affect certain disadvantaged groups [28].

Those inclined to consider responsibility for illness in allocation may wish to use predictions of *future* behaviour for reasons that parallel their endorsement of the use of past behaviours. So, a patient who is predicted to resume drinking after liver transplantation may be viewed as (in the future) responsible for the poor outcome of their transplantation (and hence be deprioritised). One challenge with holding a patient responsible for their future actions (and hence not allocating them an organ) is that this may conflict with the ‘control condition’ for moral responsibility. A patient cannot have the opportunity to control behaviour if, for example, they die from being denied an organ before the behavioural choice was even made. On the other hand, this might be thought justifiable if the prediction is based on past behaviour for which the individual *was* responsible. For example, consider if Patient B has *previously* acted in a way that increases their likelihood of not taking their medication in the future. Brown and Savulescu argue that if we assume continuity of personal identity over time, a patient who acts in a way that makes it more likely they engage in an unhealthy behaviour in the future may be prospectively responsible for that future behaviour (given that they fulfilled the conditions for responsibility at the previous time point) [31]. In theory, it may then be possible to hold Patient B responsible for their predicted future non-adherence.

However, while society is familiar with holding people responsible for past behaviour (e.g., through the criminal justice system), pre-punishment or ‘holding responsible in advance’ may appear undeserved: there is a chance the behaviour will not occur and that the punishment or holding responsible will thus itself be unfair [32]. As discussed above, this also seems particularly significant in the allocation of life-saving medical resources—where increasing a patient's chance of dying without a transplant may seem particularly harsh. Furthermore, holding patients responsible for their future actions in this manner may also have implications for patient autonomy, by infringing on their freedom to make decisions. If a patient does not receive a transplant due to their risk of certain behaviours, their autonomy is violated in that, (1) they do not receive the transplant they (presumably) desired and (2) they are presented with the consequences for their behaviour before they have been given the opportunity to control or change that behaviour. These worries appeal again to a sense of severe and undeserved punishment—and to follow the intuition that we should not punish actions that have not yet been committed.

In sum: it is difficult to justify holding patients morally accountable for actions we think they are likely to commit and deprioritise them for a liver transplant accordingly.

Nevertheless, even if we reject responsibility-based arguments (either altogether or their extension to behavioural predictions), the benefit-based argument for using behavioural predictions will remain: as long as the predictions are sufficiently accurate, society will derive more total benefit from the scarce supply of organs. While this may come at the expense of failing to properly respect certain individuals' autonomy, alternative approaches to scarce resource allocation would result in more deaths (or fewer life years saved) than we might otherwise achieve, and saving lives might reasonably be considered of overriding importance. Of course, despite best intentions, using behavioural predictions to deprioritise patients could still be *perceived* as unjustified by the public, regardless of the soundness of the underlying justification(s). As mentioned above, a lack of public acceptance poses both ethical and practical risks. Consequently, assessing public attitudes is critical. Where practical, the public should also be adequately informed about the healthcare policies that affect them so that individuals can make informed decisions accordingly.

### 2.3 Behavioural Predictions in Other Fields

Behavioural predictions are already used in at least two other areas of medicine: opioid misuse predictions in the prescribing of opioids, and risk assessments in psychiatry [4–6]. These precedents go some way towards supporting behavioural predictions in the case of liver allocation.

Opioid medications carry a high risk of dependence and misuse, placing doctors under significant professional, moral and legal pressure to prescribe these safely [33]. Consequently, assessments must be made about whether a patient is likely to misuse their prescription. Doctors can do this by checking a state drug registry [34] or using automated Prediction Drug Monitoring Programmes (PDMPs) [33]. Denying a patient request for opioids based on predicted behaviour might be justified on several ethical grounds. First, the patient's request may not be fully autonomous, i.e. addiction may affect the capacity to make decisions, which could justify a paternalistic decision not to prescribe them potentially harmful opioids. Secondly, a patient may be misleading their healthcare professional about their pain and therefore could be seen as not ‘deserving’ of the treatment. Thirdly, there may be a broader community benefit to reducing the social and healthcare system burden of opioid over-prescription and addiction.

In psychiatry, risk predictions are used to determine how likely a patient is to harm themselves or others. This may influence treatment decisions, including those which may infringe on a patient's liberties, such as submitting them to involuntary treatment or hospitalisation (which parallels the use of risk predictions in criminal justice to assist in sentencing decisions) [5, 6]. In many jurisdictions, acting on predicted behaviour is accepted when it improves outcomes for both individuals and society [35]. As with addiction, acute mental disorders often involve concerns about decision-making capacity, providing additional justification for paternalistic measures [36].

There are differences between behavioural predictions in these contexts and their use in resource allocation. Denial of opioid requests and involuntary treatment for acute mental disorders are often intended to benefit the patient. In such cases, one rationale for intervention is paternalistic. In liver allocation, the purpose of behavioural prediction is not to serve that specific patient, but rather to achieve a fair allocation of a scarce resource. The stakes of a decision involving a life-saving resource transplant may also be higher—denial of opioids or being submitted to involuntary treatment may diminish quality of life, but it is not likely to lead to a patient's death.

Important parallels remain. In both psychiatry, substance use and in resource allocation, patient benefit and patient autonomy are not the sole considerations. There are important ethical reasons potentially not to prescribe an opioid, discharge a mentally ill patient, or transplant a liver—even where the patient sincerely wishes for this to occur and would benefit from it. In each instance, one important consideration is the possibility that the decision would affect others. Insofar as the prevention of harm to others is sufficient to justify using behavioural predictions in relation to opioid use and psychiatry, there is some reason to think that it may be sufficient in relation to organ allocation as well—the individual who does not receive the liver is deprived of all possible benefit (which will occur whether a prediction is used or not), but the (mis)allocation of a life-saving transplant could also be said to harm those who would have derived greater benefit from it. Moreover, even if the prevention of harm is not sufficient to justify the use of behavioural predictions in these other areas, there may be important lessons to learn for organ allocation. We will draw on these in the discussion that follows, when we examine some potential moral differences between natural and behavioural predictions, to assess whether the latter may be problematic.

### 2.4 Can We Predict Behaviour Accurately Enough?

A potentially significant difference between behavioural predictions (Patient B) and natural predictions (Patient N) is that behaviour may be more unpredictable than biological processes. If behavioural predictions are too uncertain, their use would be harder to justify.

Accuracy is clearly critical. Early studies suggest that predicting behaviours like alcohol use and medication adherence is feasible. A preliminary AI model by Lee et al. reported an area under the curve (AUC) of 0.692 and a positive predictive value of 0.82, when externally tested to predict post-liver transplant harmful alcohol use [37], where a model is often considered ‘acceptable’ if it has an AUC of greater than 0.7 [38]. Even if this threshold is not considered sufficiently accurate for implementation, AI models are expected to become more accurate as data sets grow and the technology is refined [39]. There have also been several machine learning models published to predict medication adherence in different areas of medicine, also with promising accuracy [39].

It is difficult to ascertain how accurate a prediction needs to be for us to consider it for use. Clearly a coin flip is inappropriate, and 100% accuracy is unobtainable, but where the morally relevant threshold lies in between is unclear. One method to attempt to find this threshold is to examine predictions we currently deem acceptable for use. PDMPs currently used for opioid misuse prediction in the United States have not been validated by peer-reviewed research, and the inner workings of the algorithm used are opaque and proprietary, which makes this near impossible [4]. Furthermore, psychiatric risk assessments are notoriously unreliable, yet we also commonly use these to make decisions about patients (though not without some criticism) [40].

Importantly, tools used to predict natural events are not necessarily more accurate than proposed behavioural models. The Model for End-Stage Liver Disease (MELD) score, which predicts waiting list mortality and underpins liver allocation globally, has an AUC estimated at approximately 0.7 [41]. Despite its imperfections, many countries have accepted MELD scores as sufficiently accurate since 2002, leading to measurable improvements in outcomes [42]. If such predictions are sufficiently accurate to include in allocation, it is difficult to reject behavioural predictions on epistemic grounds.

Of course, just because MELD scores have traditionally been seen as sufficiently accurate, this does not necessarily mean that they *are* accurate enough. However, at a population level, this level of accuracy may be sufficient. Many public health policies are implemented with the aim of health improvement on a population level. For example, a malaria vaccine that is 39% effective in reducing clinical malaria rates, can reduce all-cause mortality in children in a population by 13%—a positive outcome [43]. Similarly, using a 70% accurate prediction of outcomes (whether these are behavioural or not) will increase the net benefit of the pool of livers that society has available, on a population level. The alternative—not using predictions unless they are near perfect—would lead to worse outcomes.

Another question here is whether there is a difference between epistemic requirements for predicting medical factors compared to factors involving human autonomy. One such difference could be the aforementioned difficulty predicting behaviour compared to other ‘natural’ events. As described above, this is an empirical question which may or may not be true. Secondly, the consequences could be different. One could feel more wronged if an incorrect assumption is made about one's behaviour compared to something perceived to be outside one’s control. For example, imagine patient N is told they missed out on a transplant because they were predicted to biologically reject a transplant—and new information has now come to light that this prediction was actually incorrect. Now imagine patient B is told they missed out on a transplant because they were predicted not to take their medication—but they have taken all their other medications that they are regularly on. Which patient has been more seriously wronged here?

Answers may vary. One consideration is that patient B may *feel* more wronged even if we deem the two cases to be ethically equivalent. We *feel* more in control of behaviour, and therefore we *feel* more wronged if it is used against us. This might be even more salient in the case of alcohol use. If patient B was instead told they were de-prioritised because they were predicted to relapse in their alcohol use within 5 years, but then 5 years later they remained sober—they may feel hardly done by, whereas patient N will never be exactly sure whether the prediction made about them was correct or not. These are, however, primarily issues of perception, which may be mitigated by careful implementation, informed by assessments of public attitudes and transparent policy.

There is clearly good reason to prefer more accurate predictions, particularly in high-stakes contexts such as organ allocation. We have suggested that behaviour can potentially be predicted with accuracy that is equivalent to natural predictions and will have positive outcomes on a population level, if implemented with public support.

### 2.5 Bias and Discrimination

There are two main types of wrongful discrimination: direct and indirect [44]. Direct discrimination is often said to occur when one person is treated less favourably than others on the basis that they are a member of a protected group (for example, we note that male/female gender were initially variables used in the TBS calculation but were removed after the first revision). Different views can be taken on what makes a group protected. For example, groups have been held to be protected when they are socially salient, when they have been targets of oppression or injustice previously, or when membership of the group is unchosen [35]. Paradigmatic protected groups include gender groups (or at least, non-male gender groups) and racial groups (or at least, non-White racial groups). Behavioural predictions in the context of liver transplantation would probably not—or at least need not—use protected group membership as predictive variables, so need not be directly discriminatory. However, they could still involve *indirect* discrimination.

Indirect discrimination occurs when one person a person is treated less favourably than others and the treatment, though not *based on* protected membership, is of a type that has an unjustified negative impact on members of one or more protected groups of which the person is a member [44]. Such actions are not directly discriminatory, though are similar in their effects and for that reason are often taken to be morally objectionable.

Whether they are carried out by a medical professional or an algorithm, behavioural predictions could be indirectly discriminatory. For example, predictions might negatively impact socially disadvantaged groups (e.g. certain ethnic groups) with higher rates of a range of potentially unhealthy behaviours. Here, too, behavioural predictions are not unique; other medical predictions may have similar features. For example, consider if patient N's immunological risk of rejection is genetic and related to their ethnicity (previous studies have indicated that African American patients may have a higher risk of transplant failure with some organ types) [45]. Whether this form of indirect discrimination is permissible depends on whether there is sufficient justification for the prediction and how much ethical weight we give to maximising overall benefit compared to egalitarian considerations [46].

We have two main options to reduce this type of indirect discrimination. The first option is to allocate based on a lottery or based entirely on clinical urgency. As we have outlined above, we do not find either of these strategies compelling. The second option involves making reasonable efforts to minimise differences between groups. For example, we could choose to exclude specific predictors of immune rejection, or substance abuse, if these are strongly correlated with ethnic group membership (acknowledging this may affect predictive accuracy). We should also monitor outcomes between different protected groups, to ensure these are not significantly different. While these concerns are important, they are addressable and apply equally to natural predictions; they do not discount behavioural predictions specifically.

Overall, we have argued that neither inaccuracy nor appeals to wrongful punishment and discrimination are decisive objections to behavioural predictions over natural predictions. Moreover, there are reasons to think that predicting behaviour in liver allocation would be ethically desirable, and it has some precedent in other areas of medicine.

Again, the feasibility of such a proposal depends largely on public acceptance. It is therefore important to know whether the public thinks that behavioural predictions are appropriate to use in this setting.

## 3 Part 2: Empirical Survey

### 3.1 Survey Methods

Participants were recruited through the online platform Prolific Academic. Respondents were at least 18 years of age, fluent in English, based in the United Kingdom and had a minimum Prolific approval rate of 96% (meaning that participants had submitted good quality responses to previous questionnaires). The sample was gender balanced. The survey was created using Qualtrics XM and pre-tested on colleagues and a smaller Prolific sample.

A sample size of 200 was chosen based on resource constraints. Post-hoc power analysis suggested that a sample size of 172 (accounting for excluded responses) gave us 90% power to detect medium effect sizes (*d* = 0.05) in differences between conditions at a significance level of 0.05. Precision analysis, based on a UK population size of 67 million [47], suggested that this would give a 7% margin of error at 95% confidence level.

The full survey included questions relating to the use of AI in liver allocation (reported separately) [48]. In this paper, we report results relevant to behavioural predictions (survey text in Appendix SA).

Participants were asked about behavioural predictions in two different sections.

First, participants were presented with 13 factors which could be relevant to liver allocation, partially identified from previous literature [49] and including four behavioural predictions. Participants were asked whether these factors (presented in a random order) should be used to prioritise/deprioritise waiting list patients or whether they were not relevant.

Second, participants were randomly allocated into two conditions: a ‘natural’ condition and a ‘behavioural’ condition. Participants were given a scenario (similar to the cases at the start of this paper) regarding a prediction of rejection based on immunology in the natural condition, or medication adherence in the behavioural condition (Table 1). Participants in each group were asked whether these predictions should be used to prioritise the patients.

Statistical analysis was conducted using IBM SPSS Statistics. We used descriptive statistics to measure the frequency of various responses. For comparison between natural and behavioural condition responses, Likert scales were assigned number values from 1 to 7 (where 1 = *strongly disagree*,7 = *strongly agree*), and a *t*-test was performed to compare mean scores. A *p*-value of < 0.05 was considered significant.

The project was reviewed and approved by the University of Oxford Central University Research Ethics Committee (R80692/RE003) as well as Monash University Ethics Committee (project number 34555).

### 3.2 Survey Results

Two hundred participants completed the survey. Twenty-eight were excluded for failing at least one of two attention checks (*N* = 172). The median age category was 35–44, 93.6% of respondents had completed high school or higher education, 88.4% identified as white, and 62.8% identified as having no religion (full demographics in Appendix SB).

**Figure 1:**
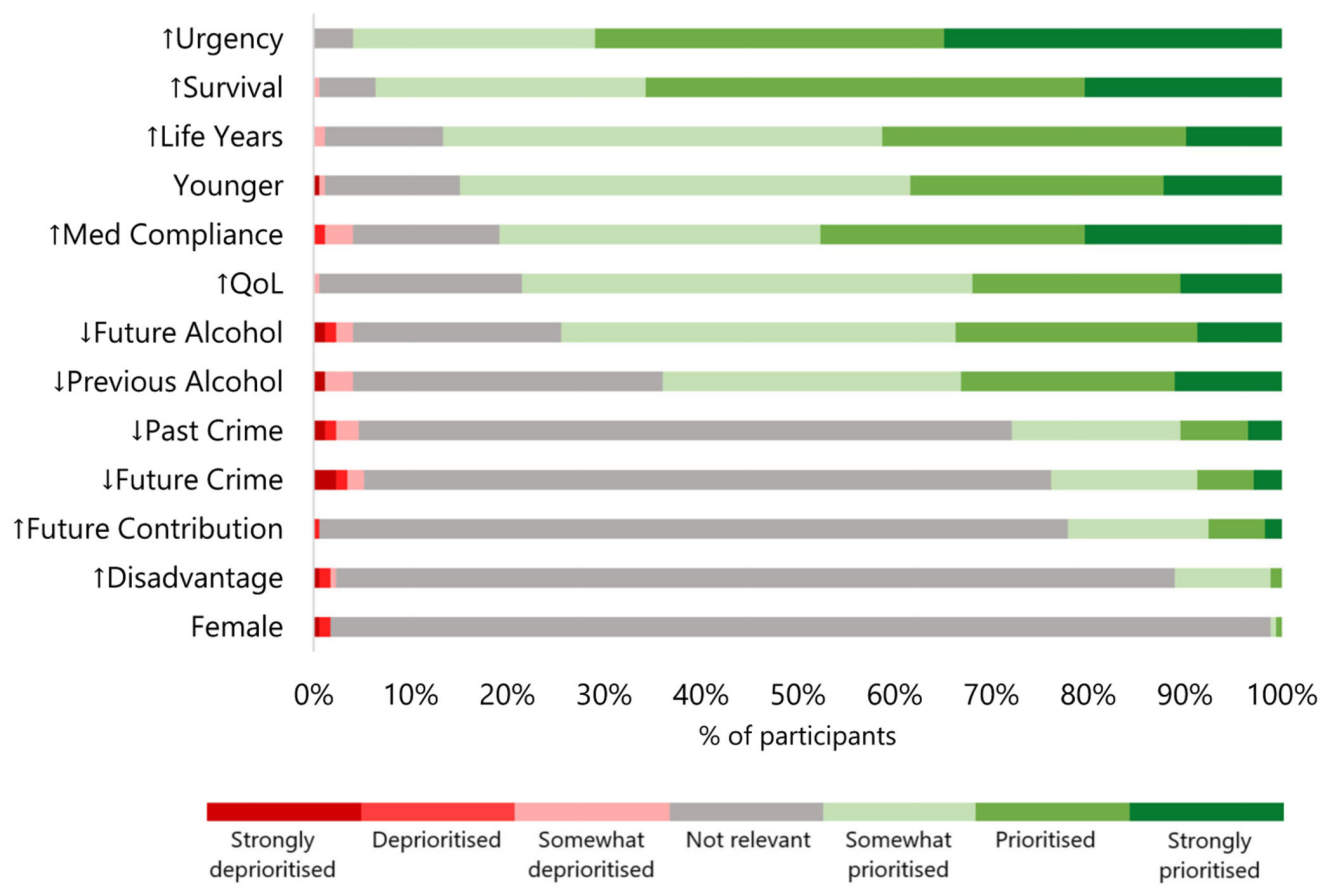
Respondent attitudes to liver allocation priority factors (*n* = 172). Each horizontal bar represents a factor which could be used in liver allocation. Green bars represent participants who thought that patients should be prioritised based on that factor (and red bars represent those who thought they should be deprioritised). Grey bars represent those who thought that factor was not relevant to liver allocation.

Most participants judged that the following characteristics should give patients priority for liver allocation: greater urgency, survival likelihood, life years gained, being younger, future medication adherence, quality of life, lower future alcohol use and lower previous alcohol use (Figure 1). On the other hand, the majority thought the following factors were not relevant to prioritisation: past crime, future crime, future societal contribution, disadvantage, and female gender. See Appendix SC for further details.

↑=increased,↓=decreased

**Figure 2:**
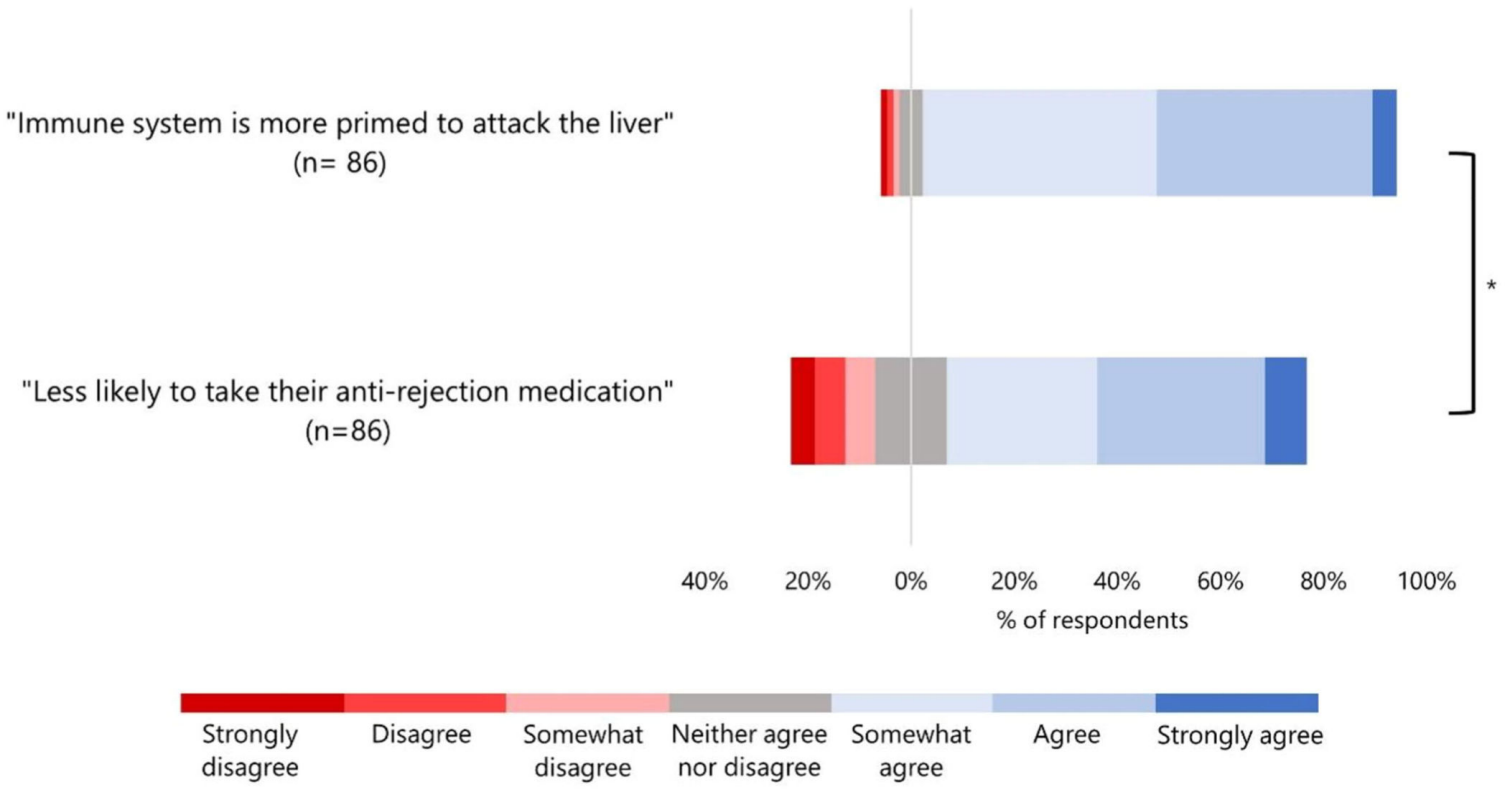
Respondent attitudes towards natural and behavioural predictions in liver allocation. Bars represents participant responses in two conditions: a patient's immune system is predicted to be more likely to attack a liver (natural condition) or a patient is predicted to be less likely to take their anti-rejection medication (behavioural condition). Red represents those who disagreed that the prediction should be used to give lower priority to that patient on the liver transplant waiting list, while blue represents those who agreed with the use of the prediction. *On average participants agreed more in the natural condition (*M* = 5.36, *SD* = 0.093) compared to in the behavioural condition (*M* = 4.87, *SD* = 1.52), *t*(141.16) = 2.54, *p* = 0.012, *d* = 0.388. Where 1 = Strongly disagree, 7 = strongly agree.

The majority of respondents agreed (strongly agreed, agreed, or somewhat agreed) that the patient with the higher chance of rejection should be deprioritised regardless of whether the prediction was immune system related or medication adherence related: 91.9% and 69.8% respectively (Figure 2). However, respondents showed *greater* levels of agreement with deprioritising this patient in the natural condition—in which the prediction was immune system-related—compared to respondents in the behavioural condition—in which the prediction was based on medication adherence (Figure 2).

### 3.3 Survey Discussion

In our survey, most respondents indicated that predictions of higher medication adherence and lower future alcohol use should be used to prioritise patients for liver allocation (78.6% and 76.5%, respectively). They appeared to support behavioural predictions when linked to medical benefit, aligning with the strong public preference for prioritising patients with the best outcomes:

92.9% responded that patients who have a higher predicted chance of survival should be prioritised. Future work could further distinguish whether participant responses on behaviour were tracking consequentialist or responsibility-sensitive views.

However, not all forms of behaviour were viewed equally. Few respondents indicated that predicted criminality and societal contribution should be used to prioritise patients (24.7% and 21.2%, respectively). This may have been because these were seen as social rather than health-related factors. Social utility was considered in some early organ allocation policies, but is now largely rejected, at least explicitly, in most jurisdictions [27].^3^ However, some argue that, in theory, non-health effects should also be considered in health policy decisions [50, 51].

In the second part of the survey, the majority (69.8%) of participants randomised into the behavioural group agreed that this prediction should be used. They would appear to support a lower priority for a patient like B in our example. Nevertheless, this was lower than the 91.9% who supported the natural prediction. Although we did not directly ask participants to compare patients N and B, the difference suggests a relative preference for natural predictions.

The reasons for this difference merit further investigation. We controlled for prediction accuracy in our survey (stipulated to be 90%). Nonetheless, it is possible that respondents (consciously or unconsciously) believe that it is not feasible to predict someone's behaviour at this level of accuracy, and therefore are less inclined to say that behavioural predictions should be used. Investigating reasons for this difference in public opinion could be the focus of future work.

Although our sample demographics roughly matched the UK population [47], and modest online convenience samples have been shown to yield similar results to representative sampling [52], further work with a larger sample size and other populations could provide more confident conclusions about the public view on behavioural predictions in liver allocation. Future surveys could also assess views on how these should be traded off against other factors in liver allocation.

Also, to note, our second question referred to the use of AI to make predictions, which could have affected responses. However, in results reported elsewhere, we did not find significant differences between attitudes towards the use of AI and human transplant committees, so we do not suspect that this significantly affected responses [48].

## 4 Conclusion

Our paper provides insights into an unexplored area of resource allocation ethics, which is likely to increase in relevance as AI predictions become more accurate.

How should we respond to the hypothetical scenario presented at the start of this paper? Should we treat patient N (natural prediction) and patient B (behavioural prediction) equally, if their outcome from transplantation is expected to be similar (and holding other factors equal)? We argue that we should. We have not identified a clear morally relevant distinction between using predictions of behavioural and natural events in liver allocation. We noted that behavioural predictions are not necessarily less accurate than natural predictions, and therefore both will increase the survival benefit that society receives from a scarce supply of organs.

The risk of indirectly discriminating against certain population groups applies to both natural and behavioural predictions. While indirect discrimination remains an important concern that should be addressed in organ allocation decisions, it does not weigh against behavioural predictions per se.

Participants in our pilot survey supported the use of behavioural predictions in liver allocation, which, if confirmed, would add democratic legitimacy to our conclusions and reduce concerns about adverse effects on donation rates. Further empirical research could explore the reasons behind public attitudes and clarify how ethical considerations inform these views.

We propose that behavioural predictions—such as medication adherence or alcohol use—should be considered alongside natural predictions, like immunological rejection. These predictions should only be used if they meet established accuracy standards (comparable to current predictive methods) and if reasonable steps are made to prevent objectionable forms of discrimination. Further work should more carefully examine how predictions of both types can be implemented in a manner that minimises harmful discrimination, particularly if algorithms and AI are used for these predictions.

Several questions remain. Even if behavioural predictions are to be used, the use of AI for this purpose raises additional issues (e.g., about bias in data sets and explainability of predictions) [53]. Furthermore, it would be important to determine how accurate we need to be when using predictions in medicine, whether they are natural or behavioural. Finally, if we accept predictions of behaviour, there are further questions about how exactly these would be implemented practically, and how they should be traded off against other ethical principles in allocation, such as urgency, responsibility, and justice.

## Supplementary Material

Appendices

## Figures and Tables

**Table 1:** Scenarios given in natural and behavioural conditions.

Natural condition	Behavioural condition
Rejection is where a patient's immune system attacks a newly transplanted liver, which may result in the liver failing. **If a patient's immune system is more primed** **to attack a new liver, this is more likely.**	Rejection is where a patient's immune system attacks a newly transplanted liver, which may result in the liver failing. **If a** **patient doesn't strictly take certain anti-rejection** **medications after transplantation, this is more likely.**
Imagine Patient X and Patient Y are both on the liver transplant waiting list, and a liver becomes available.	Imagine Patient X and Patient Y are both on the liver transplant waiting list, and a liver becomes available.
Based on a genetic test, an AI algorithm predicts that **Patient X's immune system is more primed to attack** **the liver**, and is therefore twice as likely to have rejection and the liver to fail within 6 months, compared to Patient Y. The AI prediction is known to be right 90% of the time.	Based on a genetic test, an AI algorithm predicts that **Patient X** **is less likely to take their anti-rejection medication** and is therefore twice as likely to have rejection and the liver to fail within 6 months, compared to Patient Y. The AI prediction is known to be right 90% of the time.
Indicate whether you agree or disagree with the following statement: ‘**Given the AI prediction, Patient Y should be** **prioritised over Patient X**’.	Please indicate whether you agree or disagree with the following statement: ‘**Given the AI prediction, Patient Y should be prioritised** **over Patient X**’.

## Data Availability

The data that support the findings of this study are available from the corresponding author upon reasonable request.

## Box 1 Two patients on the liver transplant waiting list.

**Table:**

Patient N: has a predicted higher chance than average (2x) of experiencing liver rejection in the first year after transplantation due to **intrinsic immunological** **factors.**	Patient B: has a predicted higher chance than average (2x) of experiencing liver rejection in the first year after transplantation due to **not adhering to their** **immunosuppressant medication regimen.**

## Footnotes

1 A strict determinist may argue that because all events are predefined by the laws of the universe (or random), behaviour is as ‘natural’ as every other event. However, we will assume here that agential events are (at least partly) within the control of an autonomous agent.

2 As previously referred to, benefit in this case refers to both minimising waiting list mortality and maximising post-transplant survival. Ideally, of course, this type of allocation algorithm would improve both.

3 Although in many European countries, priority on the kidney transplant waiting list is given to those who have previously donated a kidney, which is arguably a social utility criterion: Eurotransplant [54].
