# Supplementary material for: Should We Use Behavioural Predictions in Organ Allocation?
Source: Bioethics. 2025 Jun 29;39(8):737–47. doi: 10.1111/bioe.13440 (PMC7618015; doi:10.1111/bioe.13440)
Supplement: Supplementary file 1 — APPENDIX A. [file BIOE-39-737-s003.pdf]

APPENDIX A – SURVEY QUESTIONS

Section 1

In the first section, participants were randomised into a transplant committee condition (n=87) and an AI condition (n=85). Responses from these groups are merged in this paper as there was no practically significant difference between groups (see Appendix C).

Transplant committee condition

Preamble:

Please imagine that a liver unexpectedly becomes available for transplantation. The liver would be a match for several current patients who have severe liver failure. All the patients are currently very unwell and there is a risk they will die in the coming weeks without a transplant. Whoever receives the liver will need to take several medicines a day to stop their body 'rejecting' the transplant.

There is a need to decide which patient should receive the transplant.

Transplant committees are a group of people whose job it is to make decisions about how to allocate organs. They often include health professionals such as doctors and nurses, but also non-medical people. The committee would be told to consider one or more factors in their decisions.

We are going to ask you for your views on how transplant committees might determine which patient should receive a liver.
We will give you statements about certain factors and asking whether this means a patient should be:
Deprioritised: this should move the patient lower on the waiting list
Not relevant: transplant committees should not consider this factor
Prioritised: this should move the patient higher on the waiting list

Statements given to respondents (randomised order):

When a transplant committee allocates a liver, patients who are predicted to require a liver more urgently should be:

When a transplant committee allocates a liver, patients with a higher predicted chance of survival should be:

When a transplant committee allocates a liver, patients with more predicted years of life to gain should be:

When a transplant committee allocates a liver, patients with more predicted quality of life to gain should be:

When a transplant committee allocates a liver, patients whose liver disease was not caused by alcohol use should be:

When a transplant committee allocates a liver, patients who are predicted to be more likely to take their anti-rejection medication should be:

When a transplant committee allocates a liver, patients are sick. Please select "prioritised" to indicate that you are still paying attention. This should be:

When a transplant committee allocates a liver, patients who are predicted to be less likely to drink alcohol in the future should be:

When a transplant committee allocates a liver, patients who are predicted to be less likely to commit a future crime should be:

When a transplant committee allocates a liver, patients who are predicted to be more likely to contribute to society should be:

When a transplant committee allocates a liver, patients from a disadvantaged background should be:

When a transplant committee allocates a liver, younger patients should be:

When a transplant committee allocates a liver, patients with less of a criminal record should be:

When a transplant committee allocates a liver, female patients should be:

Response options for each statement:

- Strongly deprioritised
- Deprioritised
- Somewhat deprioritised
- Not relevant
- Somewhat prioritised
- Prioritised
- Strongly prioritised

AI condition

Preamble:

Please imagine that a liver unexpectedly becomes available for transplantation. The liver would be a match for several current patients who have severe liver failure. All the patients are currently very unwell and there is a risk they will die in the coming weeks without a transplant. Whoever receives the liver will need to take several medicines a day to stop their body 'rejecting' the transplant.

There is a need to decide which patient should receive the transplant.

Artificial intelligence (AI) might be used to allocate livers, by being programmed with one or more factors that should be considered.

We are going to ask you for your views on how AI might determine which patient should receive a liver.
We will give you statements about certain factors and asking whether this means a patient should be:
Deprioritised: the patient should be lower on the waiting list
Not relevant: AI should not be programmed with this factor
Prioritised: this patient should be higher on the waiting list

Please assume there is evidence that the AI is at least as accurate as humans.

Statements given to respondents (randomised order):

When AI is used to allocate a liver, patients who are predicted to require a liver more urgently should be:

When AI is used to allocate a liver, patients with a higher predicted chance of survival should be:

When AI is used to allocate a liver, patients with more predicted years of life to gain should be:

When AI is used to allocate a liver, patients with more predicted quality of life to gain should be:

When AI is used to allocate a liver, patients whose liver disease was not caused by alcohol use should be:

When AI is used to allocate a liver, patients who are predicted to be more likely to take their anti-rejection medication should be:

When a transplant committee allocates a liver, patients are sick. Please select "prioritised" to indicate that you are still paying attention. This should be:

When AI is used to allocate a liver, patients who are predicted to be less likely to drink alcohol in the future should be:

When AI is used to allocate a liver, patients who are predicted to be less likely to commit a future crime should be:

When AI is used to allocate a liver, patients who are predicted to be more likely to contribute to society should be:

When AI is used to allocate a liver, patients from a disadvantaged background should be:

When AI is used to allocate a liver, younger patients should be:

When AI is used to allocate a liver, patients with less of a criminal record should be:

When AI is used to allocate a liver, female patients should be:

Response options for each statement:

- Strongly deprioritised
- Deprioritised
- Somewhat deprioritised
- Not relevant
- Somewhat prioritised
- Prioritised
- Strongly prioritised

Section 2

In the second question, participants were randomised into a natural (n-86) and a behavioural (n-86) condition.

Natural condition

Rejection is where a patient's immune system attacks a newly transplanted liver, which may result in the liver failing. **If a patient's immune system is more primed to attack a new liver, this is more likely.**

Imagine Patient A and Patient B are both on the liver transplant waiting list and a liver becomes available.

Based on a genetic test, an AI algorithm predicts that **patient A's immune system is more primed to attack the liver**, and is therefore twice as likely to have rejection and the liver to fail within 6 months, compared to patient B. The AI prediction is known to be right 90% of the time.

Indicate whether you agree or disagree with the following statement:
"Given the AI prediction, patient B should be prioritised over patient A."

- Strongly disagree
- Disagree
- Somewhat disagree
- Neither agree nor disagree
- Somewhat agree
- Agree
- Strongly agree

Behavioural condition

Rejection is where a patient's immune system attacks a newly transplanted liver, which may result in the liver failing. **If a patient doesn't strictly take certain anti-rejection medications after transplantation, this is more likely.**

Imagine Patient A and Patient B are both on the liver transplant waiting list and a liver becomes available.

Based on a genetic test, an AI algorithm predicts that **patient A is less likely to take their anti-rejection medication** and is therefore twice as likely to have rejection and the liver to fail within 6 months, compared to patient B. The AI prediction is known to be right 90% of the time.

Please indicate whether you agree or disagree with the following statement:
"Given the AI prediction, patient B should be prioritised over patient A."

- Strongly disagree
- Disagree
- Somewhat disagree
- Neither agree nor disagree
- Somewhat agree
- Agree
- Strongly agree
