# Supplementary material for: Should We Use Behavioural Predictions in Organ Allocation?
Source: Bioethics. 2025 Jun 29;39(8):737–47. doi: 10.1111/bioe.13440 (PMC7618015; doi:10.1111/bioe.13440)
Supplement: Supplementary file 3 — APPENDIX C. [file BIOE-39-737-s002.pdf]

APPENDIX C

For this question, participants were originally randomised into a "transplant committee group" and an "AI group" to determine whether there was a difference between whether humans or AI used the factor in liver allocation. As there was not a practically significant difference between these (details below), we have decided to combine responses for the figure reported in the main text.

Overall, judgements did not tend to differ between the AI and transplant committee conditions in a practically significant manner. Participants tended to express slightly greater prioritisation for higher chance of survival in the transplant committee condition ($t=2.96$, $p=.003$). Participants tended to express slightly more prioritisation for those from a disadvantaged background in the transplant committee condition ($t=2.00$, $p=.048$), although on average this factor was not considered relevant. It is unclear why these differences exist, it appears possible (particularly for the disadvantaged condition) that this may indicate a type 1 error. Prioritisation judgments did not differ between conditions for any other factors. Therefore, responses from both conditions were merged.
